# Dual-wavelength photoacoustic atlas method to estimate fractional methylene blue and hemoglobin contents

**DOI:** 10.1117/1.JBO.27.9.096002

**Published:** 2022-09-01

**Authors:** Eduardo A. Gonzalez, Muyinatu A. Lediju Bell

**Affiliations:** a Johns Hopkins University, School of Medicine, Department of Biomedical Engineering, Baltimore, Maryland, United States; b Johns Hopkins University, Whiting School of Engineering, Department of Electrical and Computer Engineering, Baltimore, Maryland, United States; c Johns Hopkins University, Whiting School of Engineering, Department of Computer Science, Baltimore, Maryland, United States

**Keywords:** photoacoustic, contrast agent, monitoring, principal component analysis, coherence-based beamforming

## Abstract

**Significance:**

Methylene blue (MB) is an exogenous contrast agent that has the potential to assist with visualization and penetration challenges in photoacoustic imaging. However, monitoring the local concentration between MB and endogenous chromophores is critical for avoiding unnecessary MB accumulations that could lead to adverse effects such as hemolysis when exposed to increased dose and photodamage when exposed to high laser energies.

**Aim:**

We developed a modified version of a previously proposed acoustic-based atlas method to estimate concentration levels from a mixture of two photoacoustic-sensitive materials after two laser wavelength emissions.

**Approach:**

Photoacoustic data were acquired from mixtures of 100-μM MB and either human or porcine blood (Hb) injected in a plastisol phantom, using laser wavelengths of 710 and 870 nm. An algorithm to perform linear regression of the acoustic frequency response from an atlas composed of pure concentrations was designed to assess the concentration levels from photoacoustic samples obtained from 11 known MB/Hb volume mixtures. The mean absolute error (MAE), coefficient of determination (i.e., $R^{2}$), and Spearman’s correlation coefficient (i.e., ρ) between the estimated results and ground-truth labels were calculated to assess the algorithm performance, linearity, and monotonicity, respectively.

**Results:**

The overall MAE, $R^{2}$, and ρ were 12.68%, 0.80, and 0.89, respectively, for the human Hb dataset and 9.92%, 0.86, and 0.93, respectively, for the porcine Hb dataset. In addition, a similarly linear relationship was observed between the acoustic frequency response at 2.3 MHz and 870-nm laser wavelength and the ground-truth concentrations, with $R^{2}$ and |ρ| values of 0.76 and 0.88, respectively.

**Conclusions:**

Contrast agent concentration monitoring is feasible with the proposed approach. The potential for minimal data acquisition times with only two wavelength emissions is advantageous toward real-time implementation in the operating room.

## 1 Introduction

Exogenous chromophores^1^^,^^2^ play a critical role in photoacoustic drug delivery,^3^^,^^4^ overcoming imaging challenges associated with visualizing low signal amplitudes, small vasculature targets, and deep structures that suffer from optical and acoustic attenuation. Over the past 20 years, exogenous chromophores have been administered to increase signal contrast with beneficial applications in angiogenesis for cancer monitoring,^5^^,^^6^ enhanced photoacoustic angiography,^7^ lymph node tracers in breast cancer,^8^^,^^9^ deep imaging,^10^ lymphatic drainage,^11^ and brain imaging.^12^ Possible contrast agent chromophores include gold nanostructures,^5^^,^^13^^,^^14^ carbon nanotubes,^15^ fluorescent proteins,^11^^,^^16^^,^^17^ methylene blue (MB),^18^^,^^19^ and indocyanine green (ICG)^7^^,^^20^^,^^21^ dyes.

Depending on the dose, exposure time, and number of target cells, adverse effects due to misuse of contrast agents include acute inflammation,^22^ apoptosis,^22^^,^^23^ necrosis,^14^ cellular toxicity,^24^ allergies,^25^ reduction in cellular viability,^26^ nephropathy,^27^ hemolysis,^28^ and photodamage.^29^ In addition, maximum absorption and clearance times (i.e., the time for the drug to completely leave the system) range from 5 min to 24 h among the drug delivery approaches reported in the literature,^20^^,^^30^^–^^32^ requiring different dose, delivery, and monitoring protocols.

Currently, most approaches to measuring the concentration levels of chromophores rely on the acquisition of photoacoustic responses from multiple laser wavelength emissions paired with spectral unmixing methods.^33^^–^^40^ However, as real-time implementations are necessary for monitoring chromophores with short clearance times in applications such as photoacoustic-guided surgery,^41^^–^^44^ traditional techniques are typically not feasible because of the lengthy acquisition times associated with transmitting multiple laser wavelengths to achieve a single estimate.^45^ In addition, traditional spectral unmixing techniques do not typically consider differences in acoustic spectra, which has the potential to provide additional information for differentiation of exogenous and endogenous chromophores.

We previously proposed an acoustic frequency-based method to discriminate photoacoustic responses from different materials and overcome challenges with traditional spectral unmixing techniques.^46^ By measuring the photoacoustic response from only two laser wavelengths, the initial version of our dual-wavelength atlas method achieved comparable sensitivity, specificity, and accuracy to traditional spectral unmixing methods^47^^,^^48^ and related classifiers.^49^^,^^50^ Based on the linear relationship between contrast agent concentration and photoacoustic amplitude,^51^^–^^53^ we hypothesize that our method can be extended to locally characterize the volumetric ratio between two photoacoustic-sensitive materials. Specifically, we focus on the exogenous chromophore MB and the endogenous chromophore hemoglobin (Hb), motivated by the potential for photoacoustic-based catheter interventions performed with an optical fiber housed within a cardiac catheter inserted through a major vein.^54^ MB would be administered through the catheter and eventually mix with Hb to enhance the visualization of target structures in vascular pathologies (e.g., atherosclerositic plaques,^55^ thrombi,^56^ tumor neovasculatures,^57^^,^^58^ and endothelia^59^), and the local concentration of MB versus Hb would be monitored with our proposed method with either external^54^ or intravascular^60^ ultrasound sensor placement. This monitoring will enable real-time interventions to avoid the adverse effects produced by the unnecessary accumulation of MB.^61^^–^^64^ In addition, the MB used in the present study serves as a surrogate for other potential intravascular contrast agents that would similarly benefit from local concentration monitoring to prevent cell toxicity.^24^^,^^60^

The remainder of this paper is organized as follows. Section 2 details the phantom experimental setup, a novel dual-wavelength atlas method to estimate MB and Hb concentrations, and quantitative metrics for performance evaluation. Section 3 presents the quantitative evaluation of mixture estimation performance, as well as the evaluation of linear and monotonic trends of estimated concentration versus ground-truth labels, including an assessment of appropriate region sizes to perform the proposed estimation. Section 4 discusses insights from these results, and Sec. 5 summarizes the clinical impact of the proposed methods.

## 2 Method

### 2.1 Experimental Setup

**Fig. 1:**
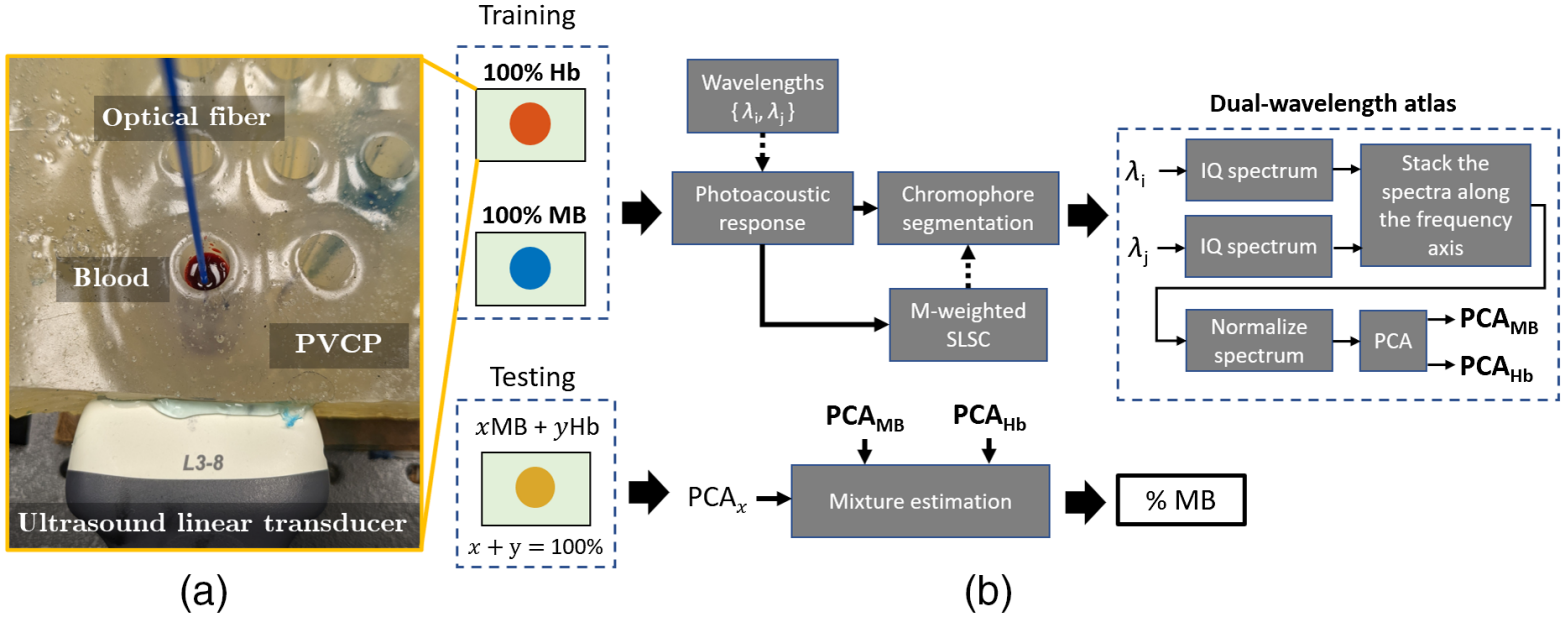
(a) Experimental setup and (b) framework of the dual-wavelength atlas method for mixture estimation of MB and Hb.

A polyvinyl chloride-plastisol (PVCP) phantom was fabricated with hollow chambers, each with a diameter of 15 mm and a depth of 55 mm. To minimize the variability of ultrasound scattering among chambers due to different air bubble distributions, a single chamber was filled with either a 100-μM aqueous solution of MB from Fisher Scientific (Waltham, Massachusetts), blood (Hb) or a combination of MB and Hb. A 1-mm-diameter optical fiber was inserted in the filled chamber, and the fiber tip was positioned ∼20  mm below the top surface. The optical fiber was connected to a Phocus Mobile laser (Opotek Inc., Carlsbad, California), transmitting laser light with wavelengths of 710 and 870 nm and with a laser energy of 3 mJ. The resulting photoacoustic signals were received by an Alpinion L3-8 linear array ultrasound probe (Alpinion Medical Systems, Seoul, South Korea) positioned on the lateral wall of the phantom, ∼20  mm from the hollow chamber cross section. This experimental setup, which is shown in Fig. 1(a), has been described in previous publications.^46^^,^^65^

Two types of Hb samples were used in this study. First, 23 vials of fresh human Hb were obtained up to two days after blood draw and storage, mixed in a single container, and exposed to air for ∼1  h to minimize the variability of Hb oxygenation levels. Second, experimental whole porcine blood (Innovative Research, Novi, Michigan) was used on the fourth day of its reported 24-day lifetime.

A total of 11 concentration levels were prepared using different 2-mL mixtures of Hb and MB. These concentrations ranged from 0% to 100% in 10% increments of MB volume percentage. For example, for the 2-mL MB-Hb mixture, a concentration of 60% MB consisted of 1.2 mL of 100-μM MB and 0.8 ml of Hb. Each concentration mixture was manually stirred with a syringe in a separate container. Unless otherwise stated, the % concentrations reported in this manuscript refer to the % MB concentration. Five trials were conducted for each concentration level by fixing the light-delivering optical fiber at 0 deg, with 20 photoacoustic frames (10 per wavelength) acquired per trial using a frame rate of 5 Hz. It is worth noting that two blocks of 64-channel aperture data were required to obtain an image created from 128 receive elements, which reduced the possible frame rate from 10 Hz (based on the 10-Hz laser pulse repetition frequency) to 5 Hz.

### 2.2 Dual-Wavelength Atlas Method for Mixture Estimation

The framework for mixture estimation is shown in Fig. 1(b), based on the dual-wavelength atlas method previously proposed for the identification of individual chromophores.^46^ With this setup, frequency domain information is expected to be useful because the optical fiber is placed inside the PVCP chamber, thus generating fluence maps that diminish radially from the tip of the optical fiber. This fluence distribution generates unequal acoustic frequency responses, as volume regions of different sizes are being excited. Thus, the proposed algorithm is anticipated to leverage frequency domain information to differentiate photoacoustic responses from various concentrations of chromophores.

To implement the proposed approach, conventional delay-and-sum (DAS) beamforming was first employed to create photoacoustic images for each wavelength emission, concentration, and trial. In contrast to preceding work,^66^ we used M-weighted short-lag spatial coherence (SLSC)^67^ with a cumulative lag M=20 instead of conventional SLSC with M=5 to generate the binary masks that segmented signals of interest and provided ground-truth labels. This specific M-weighted SLSC cumulative lag value (i.e., M=20) was chosen based on the comparison of SLSC^68^ images with M-weighted SLSC. First, a representative SLSC^68^ photoacoustic image from the blood dataset was generated with M=5. The same photoacoustic frame was then processed with M-weighted SLSC with M values ranging from 10 to 30. Binary masks generated with a −3-dB threshold were then created for each image, and the similarity was defined with the Dice coefficient:^69^

$$\text{Dice coefficient}=\frac{2\sum \mathrm{AND}({\text{Mask}}_{\mathrm{SLSC}},{\text{Mask}}_{M\text{-Weighted }\mathrm{SLSC}})}{\sum {\text{Mask}}_{\mathrm{SLSC}}+\sum {\text{Mask}}_{M\text{-Weighted }\mathrm{SLSC}}}\times 100\%,$$ (1)

where AND is the logical “AND” operator between the binary masks. This process was then repeated for one trial of the human Hb dataset, containing 11 concentrations, 2 wavelengths, and 10 frames. The Dice coefficients calculated between SLSC and M-weighted SLSC masks were then plotted as a function of M, resulting in M=20 yielding the highest mean similarity to the SLSC masks with the least standard deviation.

The modification from SLSC in our preceding work^66^ to M-weighted SLSC in this work was implemented to increase the influence of lower lags over higher lags, thus creating less disjointed binary masks. A single binary mask was obtained per trial, resulting from the logical inclusive “OR” operation of 20 masks (obtained from 10 frames per two laser wavelengths).

As noted in Fig. 1(b) and in our previous work,^66^ for each image, only those pixels included in the coherence masks were used for feature extraction, training, and classification. In-phase and quadrature (IQ) demodulation was implemented with 2.75 MHz and 85% bandwidth, and the resulting analytic spectra from a sliding window of axial kernels was stacked along the frequency axis. For the last step of feature extraction, principal component analysis (PCA) was applied to the power of the stacked spectra to reduce the complexity of the feature space.

The training dataset consisted of a random trial of 0% MB (i.e., 100% Hb) and 100% MB, and the remaining trials and concentration levels were included in the testing datasets. After the dual-wavelength atlas was constructed [i.e., ${\mathrm{PCA}}_{\mathrm{MB}}$ and ${\mathrm{PCA}}_{\mathrm{Hb}}$ in Fig. 1(b)], a mixture estimation algorithm was designed to obtain the concentration level of MB from a testing sample. First, N=1000 samples were randomly selected from the MB and Hb atlas. Then, assuming that mixtures are linear combinations of pure MB and Hb concentrations, a concentration distribution $C^{\prime }\in R^{N\times 1}$ was obtained using the following equation:

$$C_{x}^{\prime }=\frac{1}{2}+\frac{{\left\|{\mathrm{PCA}}_{x}-{\mathrm{PCA}}_{\mathrm{Hb}}\right\|}_{1}-{\left\|{\mathrm{PCA}}_{x}-{\mathrm{PCA}}_{\mathrm{MB}}\right\|}_{1}}{2{\left\|{\mathrm{PCA}}_{\mathrm{Hb}}-{\mathrm{PCA}}_{\mathrm{MB}}\right\|}_{1}},\;0\leq C^{\prime }\leq 1,$$ (2)

where ${\mathrm{PCA}}_{x}$ is the projected spectra of x% testing concentration, ${\left\|.\right\|}_{1}$ is the Manhattan norm, and each PCA matrix is of size $R^{N\times p}$ with p principal components. A histogram filter was then applied to the $C^{\prime }$ vector, where the bin with the highest number of individuals out of 10 bins was extracted. The mean value $C^{\prime \prime }$ was computed from the reduced $C^{\prime }$ vector, and the process was repeated for each valid pixel and frame of the trial. Finally, a median filter was applied to the concentration maps with a kernel of 0.64×0.70  mm×30 in the axial, lateral, and frame dimensions, respectively. The 0.64-mm axial kernel length was selected to reside within the frequency response of the received photoacoustic signals, which ranged from 2 to 2.75 MHz, corresponding to wavelengths ranging from 0.77 to 0.56 mm, respectively. As the lateral and axial resolutions of an image differ, the lateral kernel width (i.e., 0.70 mm) was chosen to be the closest possible match to the axial kernel length. The frame dimension of the kernel reduced the concentration tensor from $C^{\prime \prime }\in R^{N_{i}\times N_{j}\times N_{f}}$ to $C\in R^{N_{i}\times N_{j}}$, where $N_{i}$ and $N_{j}$ are the number of rows and columns, respectively, of the reconstructed photoacoustic image.

Because Eq. (2) is applied on frequency data, the three most influential parameters of the dual-wavelength atlas method for the estimation of C are the axial kernel size, threshold of spectral log compression, and number of principal components. An increase in axial kernel size improves the accuracy of the estimation of spectral amplitudes in the Fourier space. The threshold of spectral log compression adds a tolerance to the discrimination between spectral peaks, which affects the variance that is computed in the PCA. The number of principal components controls the amount of information that is reduced to the feature space.

To provide complementary information regarding the location of photoacoustic targets in the imaging plane, the displayed concentration maps were overlayed with an ultrasound image of the plastisol chamber processed with locally weighted SLSC (LW-SLSC),^70^ using a regularization factor α=1 and a 1.25  mm×1.2  mm kernel. The LW-SLSC parameters were obtained from our previous optimization study.^66^ A factor of α=1 corresponded to an equal weight between cost and penalty function for calculating the optimal coefficients in LW-SLSC. An axial kernel size of 1.25 corresponded to approximately 4.5λ, where λ is the wavelength associated with the center frequency of the L3-8 ultrasound probe.

### 2.3 Quantitative Metrics

To evaluate the overall accuracy of the dual-wavelength atlas method for mixture estimation, the estimated concentration levels C were first ordered as a function of the ground-truth labels k. The function $f_{1}:C(k)$ was then compared with $f_{2}:C=k$, representing a 1:1 relationship between estimated and true concentrations. Finally, the coefficient of determination $R^{2}$ was used to quantify the performance of the dual-wavelength atlas method:

$$R^{2}=1-\frac{\underset{k}{\sum }\underset{i}{\sum }C_{ki}-k}{\underset{k}{\sum }\underset{i}{\sum }C_{ki}-\overset{¯}{C}},$$ (3)

where $C_{ki}$ is the estimated concentration of measurement i for a ground-truth concentration k and $\overset{¯}{C}$ is the mean estimated concentration. In addition, the $R^{2}$ metric was used to assess the linearity of the photoacoustic spectra obtained from mixtures of MB and Hb by implementing a linear regression of spectral amplitudes as a function of the ground-truth concentrations.

Similarly, monotonicity was evaluated in two cases: (1) between photoacoustic spectral amplitudes of a specific frequency and ground-truth concentrations and (2) between estimated and ground-truth concentrations. The monotonic trend of these data pairs were quantified with Spearman’s rank correlation coefficient ρ, described in the following equation:^71^

$$\rho =\frac{\mathrm{cov}(R(X),R(Y))}{\sigma_{R(X)}\sigma_{R(Y)}},$$ (4)

where R(X) and R(Y) are the rank variables of the measurements X and Y, respectively, X is either the spectral amplitudes or estimated concentrations, Y is the ground-truth concentration, cov represents covariance, and σ is the standard deviation of the rank variables. Spearman’s ρ values of 1 and −1 represent perfect monotonic trends between measurements X and Y that are increasing and decreasing, respectively. It is worth noting that Spearman’s ρ measures the level of monotonicity, rather than determining whether or not an evaluated function is monotonic. In this study, we characterize |ρ|≥0.8 as a strong monotonic trend.

The mean absolute error (MAE) was used to assess the accuracy of the generated concentration maps:

$${\mathrm{MAE}}_{k}=\frac{\overset{N_{k}}{\underset{i=1}{\sum }}|C_{ki}-k|}{N_{k}},$$ (5)

where $C_{ki}$ is the estimated concentration of pixel i for a ground-truth concentration k and $N_{k}$ is the total number of pixels of the concentration map k.

Finally, processing times were computed for each stage of the dual-wavelength atlas method applied on the human Hb dataset. These stages were (1) generation of coherence masks, (2) generation of acoustic spectra and spectra stacking, (3) PCA, and (4) estimation of MB concentrations, with the last stage including projection to the feature space using the principal component coefficient obtained from the atlas. These processing times were evaluated individually when training our method (i.e., constructing the atlas) and testing our method on a single frame (i.e., estimating a concentration map). The processing times for stages (3) and (4) were only calculated for training and testing, respectively. For training, robustness in the estimation of computation times was achieved by averaging the processing times from 5 different trials of 0% concentration. Similarly, robustness in the estimation of computation times for testing was achieved by averaging the processing times from every frame of the testing dataset detailed in Sec. 2.2. Computation times were measured using the MATLAB (Natick, Massachusetts) environment executed on an Intel Core i5-6600K processor with 32 GB RAM and a TITAN Xp graphical processing unit (GPU). To enhance the computation speed of stage (1) for all results reported in this manuscript, M-weighted SLSC was implemented on the GPU described above, following the architecture of preceding work.^68^ In addition, this GPU-M-weighted SLSC approach and the previously-reported GPU-SLSC approach^68^ were both implemented to obtain results for Eq. (1) in Sec. 2.2.

## 3 Results

### 3.1 Concentration Estimations from MB and Human Hb

**Fig. 2:**
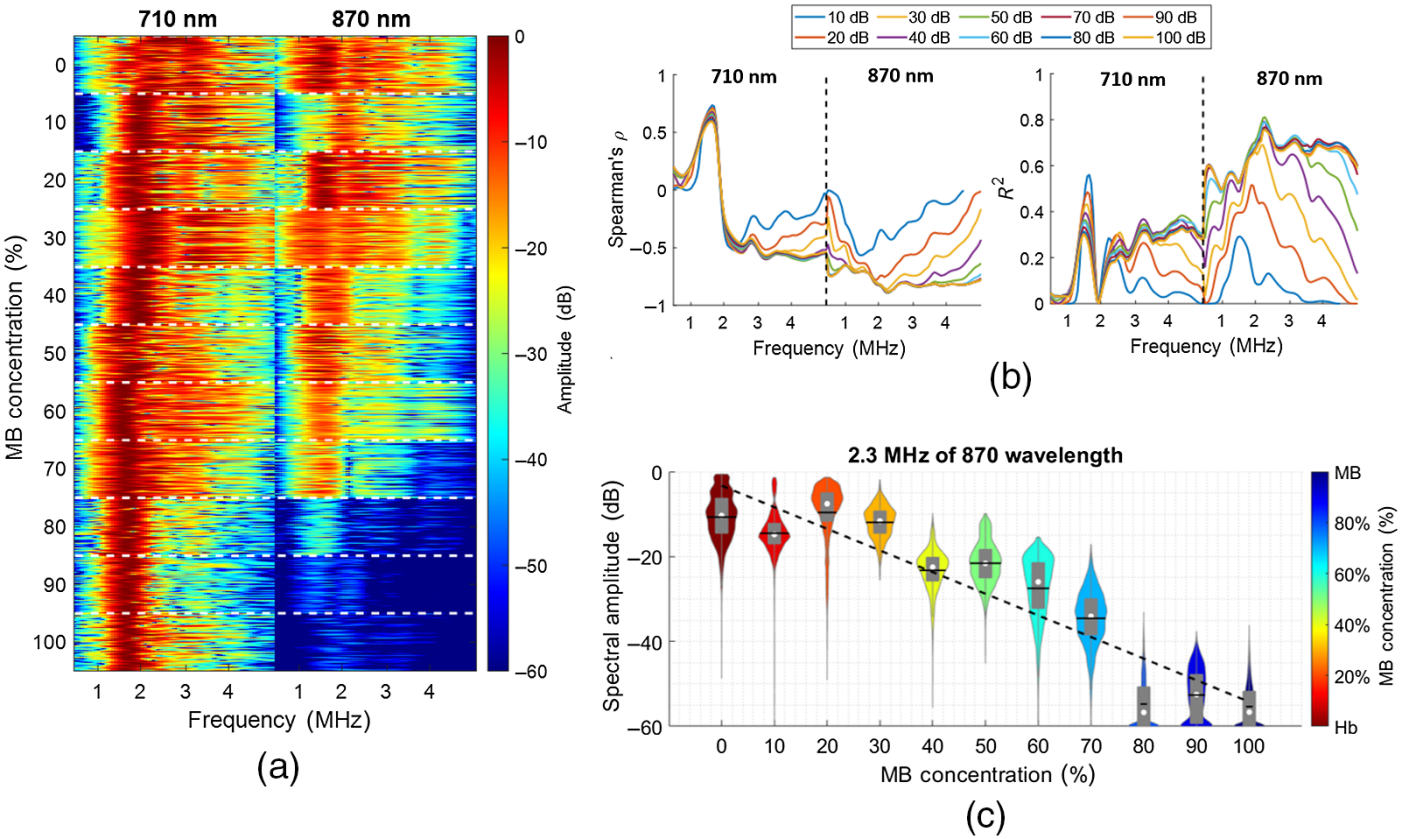
Evaluation of monotonicity and linearity of photoacoustic spectra obtained from mixtures of MB and Hb. (a) Stacked photoacoustic spectra of several mixture concentration (y axis) when using 710- and 870-nm laser wavelengths (x axis). Each spectrum is normalized and log compressed to a 60-dB dynamic range. (b) Spearman’s ρ coefficient and $R^{2}$ values of linear regression of concentration levels versus acoustic frequency while varying the log compression dynamic range of the acoustic spectra. (c) Example of an acoustic frequency with a spectral amplitude of concentration levels that follow a monotonic trend (ρ=−0.88) and a linear trend ($R^{2}=0.76$).

Figure 2 shows the results from the photoacoustic spectra obtained with mixtures of MB and human Hb. Stacked photoacoustic spectra are shown in Fig. 2(a) with the y axis denoting mixture concentration and the x axis denoting acoustic frequency for optical wavelengths 710 (left) and 870 nm (right). Each concentration block separated by the dashed white lines in Fig. 2(a) shows 20,000 spectra samples that were randomly selected from the total number of training trials, frames, and kernels. Although a subtle frequency shift is observed in the spectra obtained with the 710-nm wavelength when increasing the MB concentration level, the spectral shift across the mixture for the 710-nm response was not strongly monotonic (ρ=−0.63). Similarly, no apparent spectral shift was observed for the 870-nm response (ρ=−0.27). One possible cause of the frequency shift in the spectra obtained with the 710-nm wavelength is the photoacoustic interaction with residual particles of MB in the PVCP chamber. These particles were unable to be removed as they stained the chamber walls, and they likely added frequency components to the overall spectral response of different chromophore concentrations.

Figure 2(b) shows the linearity and monotonicity evaluation results as a function of the acoustic frequency for multiple image dynamic ranges. The y axes denote the ρ (left) and $R^{2}$ (right) for and linearity and monotonicity evaluations, respectively. The x axis of each plot is divided into acoustic frequencies for optical wavelengths of 710 nm (left) and 870 nm (right). In the acoustic frequency range of 0.5 to 5 MHz for the 870-nm wavelength, as the dynamic range decreases from 40 to 10 dB, the mean ± one standard deviation of improvements in Spearman’s ρ and $R^{2}$ were −0.47±0.15 and 0.45±0.14, respectively. For dynamic ranges >40  dB, the results primarily overlap, and there is less improvement. This overlap likely occurs because the log compression reaches the resolution limit of the acquired photoacoustic amplitudes. However, for the 710-nm wavelength, only the acoustic frequency range from 2.4-5 MHz shows ρ and $R^{2}$ changes when varying the log compression from 10 to 40 dB. In the acoustic frequency range of 0.5 to 2.4 MHz, results primarily overlap regardless of the chosen dynamic range. This overlap likely occurs because of the strong spectral peak observed from 1.5 to 2.5 MHz for nearly every MB concentration in Fig. 2(a) (i.e., the spectral shift pattern described in the preceding paragraph). This strong spectral peak is robust against log compression, which generates minimal changes to Spearman’s ρ and variation of $R^{2}$.

Focusing on the $R^{2}$ results, multiple strong linear trends were observed when results were obtained with 870-nm laser wavelength, yielding a mean ± one standard deviation $R^{2}$ of 0.74±0.14 for frequencies ranging from 0.5 to 5 MHz and thresholds from 10 to 100 dB. In contrast, no strong linearity was observed for the 710-nm region, yielding an $R^{2}=0.32\pm 0.21$ for the same range of frequencies and thresholds as the 870-nm region. The strong spectral peak observed in Fig. 2 at the 710 nm wavelength yielded a maximum $R^{2}$ of 0.55 with a dynamic range of 10 dB, whereas the maximum peak observed at 870 nm was 0.81 with a dynamic range of 50 dB. These results suggest that Hb has a stronger influence on the linear relationship of the acoustic frequency versus concentration compared with MB.

Figure 2(c) shows the linear fit for the acoustic frequency of 2.3 MHz and the dynamic range of 60 dB [i.e., parameters that produced the maximum $R^{2}$ in Fig. 2(b)]. The y axis denotes the log-compressed spectral amplitudes, and the x axis denotes the ground-truth concentrations. For each violin plot, the shape, horizontal line, white circle, and gray box represent the kernel density estimate of the data points, mean, median, and interquartile range, respectively. $R^{2}=0.78$, suggesting a strong linearity and monotonicity between the spectral amplitudes of photoacoustic signals and ground-truth concentrations. Spearman’s ρ was 0.89, which indicates that there is a high level of monotonicity.

**Fig. 3:**
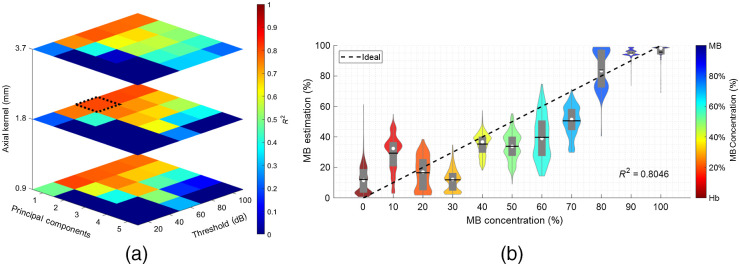
Performance optimization of the dual-wavelength atlas algorithm using the human Hb dataset. (a) $R^{2}$ values as a function of axial kernel size (z axis), threshold for spectra log compression (x axis), and number of principal components used (y axis). (b) Examples of mixture estimations (y axis) versus ground truth concentrations (x axis) when using the parameter set that yielded the highest $R^{2}$ value (i.e., 0.80), represented by the dotted square in (a).

Figure 3 shows the optimization of the dual-wavelength atlas method using the human Hb dataset by maximizing the $R^{2}$ fit of a 1:1 slope of estimated versus ground-true concentrations. The variation of $R^{2}$ values as a function of the three most influential parameters of the dual-wavelength atlas method is shown in Fig. 3(a). The z axis denotes the axial kernel size, and the orthogonal axes denotes the number of principal components used and the threshold for spectra log compression, respectively. Although increasing the axial kernel size improved the accuracy of the spectral amplitude generated with fast Fourier transforms, no clear improvement was observed when measuring the overall $R^{2}$ values. Similarly, parameter sets using two principal components yielded $R^{2}\geq 0.7$, suggesting a good estimation performance when using more than one principal component. However, no improvement was observed for PC≥3. Finally, increasing the threshold from 20 to 60 dB improved the $R^{2}$ values when other parameters remained fixed, and no additional improvement was observed for dynamic range thresholds ≥60  dB.

Figure 3(b) shows an example of concentration estimation versus ground-truth concentration when using the optimal set of parameters that yielded the greatest $R^{2}$ value, denoted by the dashed black box in Fig. 3(a). For this optimized estimation result, the $R^{2}$ value and Spearman’s ρ were 0.80 and 0.89, respectively, indicating a high degree of linearity and monotonicity, respectively.

### 3.2 Concentration Estimations from MB and Porcine Hb

**Fig. 4:**
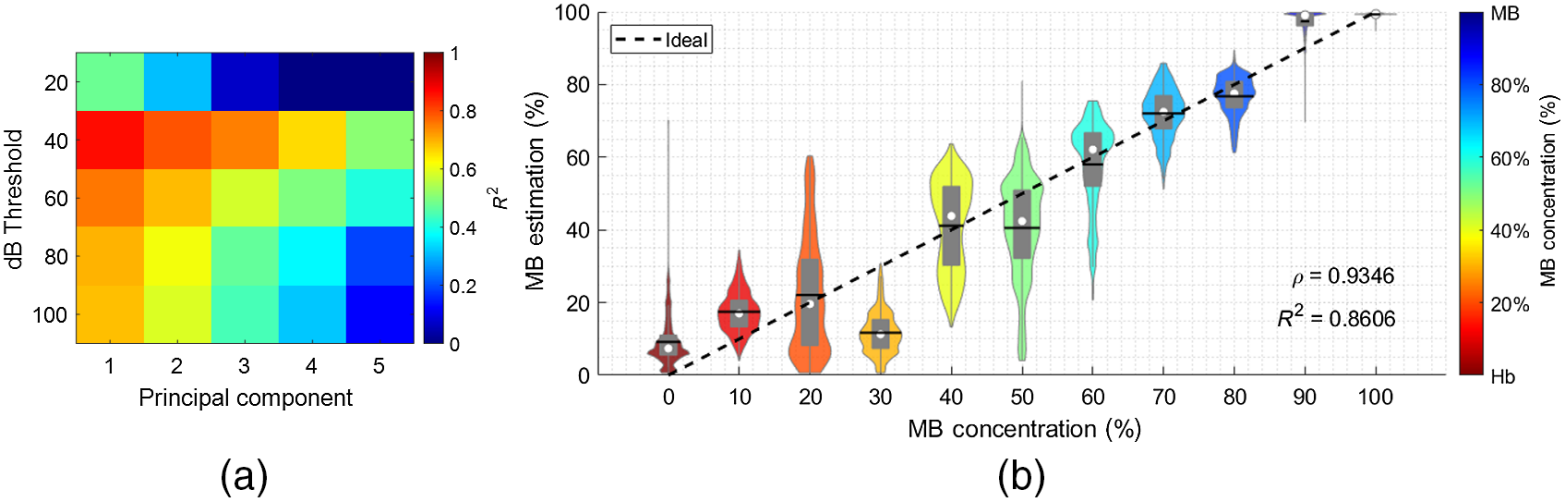
Estimated concentrations from porcine Hb. (a) $R^{2}$ value of a 1:1 linear fit between estimated and true concentration levels while varying the threshold for spectra log compression (y axis) and the number of principal components used (x axis). (b) Mixture estimation (y axis) versus ground truth concentration (x axis) when using a 40 dB threshold and 1 principal component.

Figure 4 shows the estimated concentrations when using porcine Hb. The sensitivity of estimated $R^{2}$ values while varying two parameters of the dual-wavelength atlas method is shown in Fig. 4(a) with the y axis denoting the threshold for spectra log compression and the x axis denoting the number of principal components used. These $R^{2}$ values were computed by fixing the axial kernel size to 1.8 mm [i.e., optimal parameter obtained in Fig. 3(a)]. Overall, the effect of varying the number of principal components and the dB threshold implemented prior to estimating the $R^{2}$ values showed similar trends to those observed in Fig. 3(a) (i.e., decreasing estimation performance with increasing the number of principal components). However, the optimal set of parameters included a threshold of 40 dB instead of the 60 dB threshold obtained in the experiment using human Hb [i.e., Fig. 3(a)], with $R^{2}=0.86$ and ρ=0.93.

Figure 4(b) shows detailed estimation results when using the optimal set of parameters from Fig. 4(a). The overall performance of the dual-wavelength atlas method when using the porcine Hb was measured by computing the MAE between the vector constructed with the mean of each violin plot and the ground-truth labels, yielding a value of 6.53%. For comparison, the MAE obtained from the optimal parameter set using the human Hb [i.e., Fig. 3(b)] was 10.49%. Thus, the dual-wavelength atlas method generated more accurate estimates of MB concentration when using porcine Hb instead of human Hb, which can be attributed to the uniformity in chemical composition and oxygenation levels of the porcine Hb samples.

**Fig. 5:**
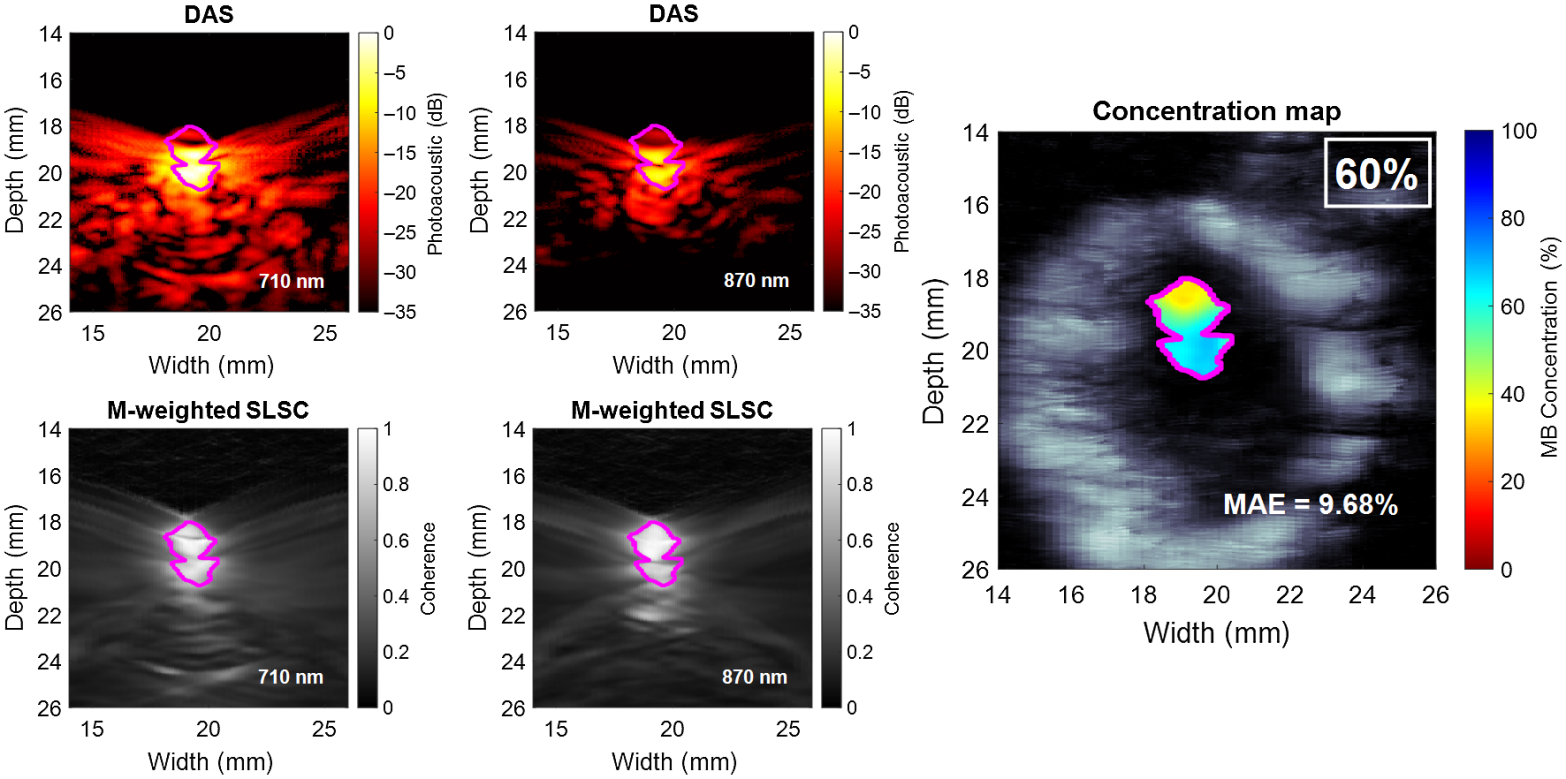
Example of photoacoustic DAS images, coherence masks used for segmentation, and estimated concentration map for a ground-truth concentration of 60% (i.e., 60% MB and 40% experimental porcine Hb). The purple contours represent the masks obtained by thresholding the coherence images at –3 dB and merging the results from 710 and 870 nm.

Figure 5 shows example DAS photoacoustic images, coherence masks, and an estimated concentration map for a ground-truth concentration of 60% of a single trial. These DAS images were normalized to the maximum amplitude value obtained from both 710- and 870-nm laser wavelength responses and displayed with 35-dB dynamic range to offer a direct comparison across wavelengths. The contours within each image represent the −3  dB boundary of the coherence mask with an area of $5.32\;{\mathrm{mm}}^{2}$. It is worth noting that these masks include the low-amplitude regions of the photoacoustic images that appear if minimal signal is present, which highlights the benefit of the coherence mask that is used to determine the presence of coherent signals, regardless of amplitude. The MAE between the concentration map and the ground truth is 9.68%.

Table 1 reports the average processing times for each stage of the dual-wavelength atlas method using the porcine Hb dataset. Without considering acquisition times and transferring times of raw photoacoustic data, the construction of the dual-wavelength atlas was completed in <6  min, and the mean ± one standard deviation processing time of a single concentration map was 9.2±0.5  s. This <10  min processing time is relatively fast in comparison with the total procedure times of cardiovascular interventions (e.g., the average procedure time of perfusion coronary interventions is 76±31  min^72^), and it can be performed prior to the initiation of the procedure.

**Table 1:** Processing times (in seconds) for each training and testing stage of the dual-wavelength atlas method.

Stage	Training	Testing (one frame)
Generate coherence masks	135.5 ± 0.4 s	2.3 ± 0.1 s
Convert to frequency domain	180.9 ± 23.6 s	6.4 ± 0.8 s
Apply PCA	2.6 ± 0.1 s	—
Estimate concentrations	—	0.6 ±0.2 s
Total	319.2 ± 23.7 s	9.2 ± 0.5 s

**Fig. 6:**
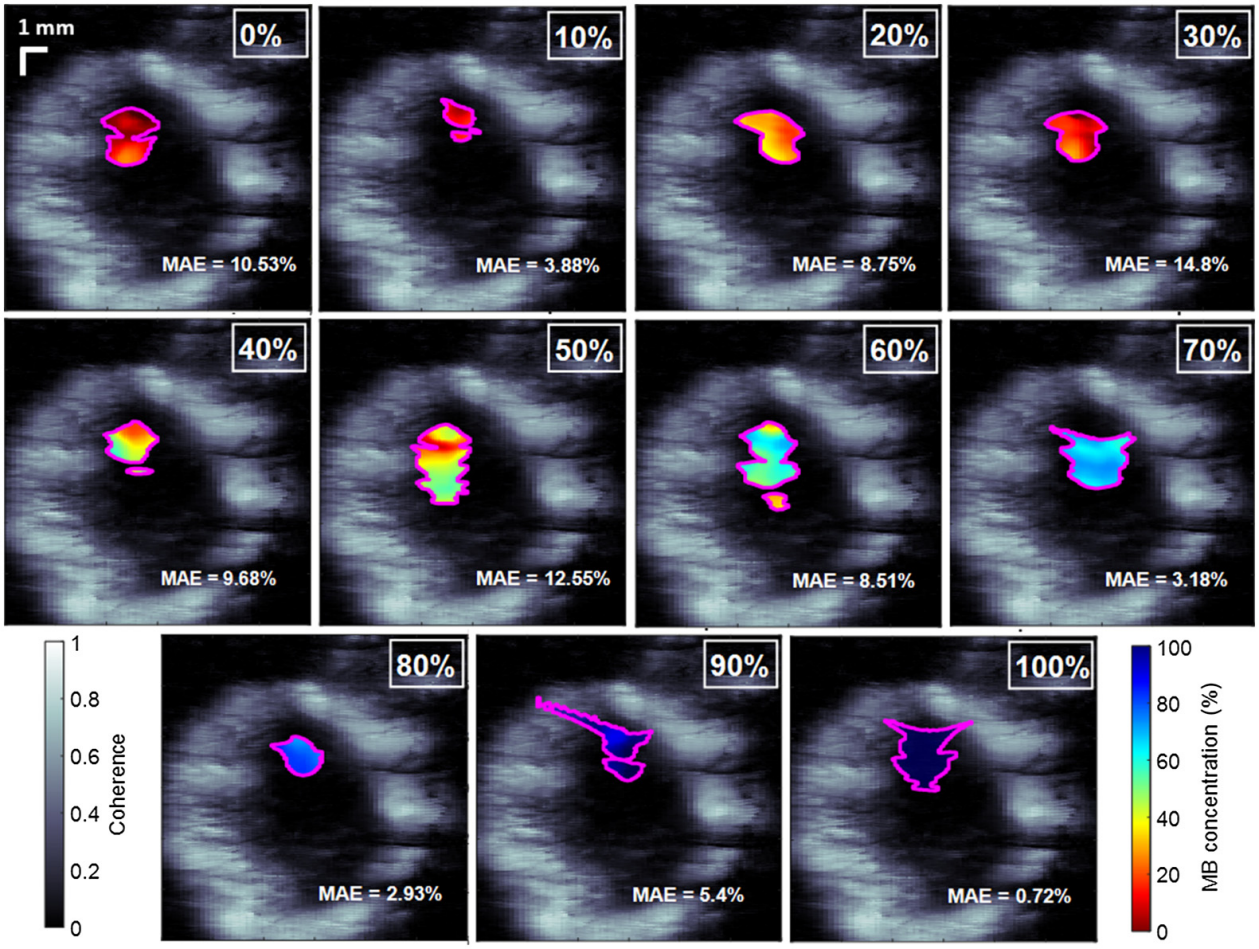
Examples of concentration maps of MB and porcine Hb generated by the dual-wavelength atlas method in a phantom experiment. The concentration maps (colored) are overlaid on ultrasound images generated with LW-SLSC.

Figure 6 shows examples of concentration maps obtained from one trial per ground-truth concentration results. The 30% concentration map produced the greatest MAE of 14.80%, whereas the 100% concentration maps produced the lowest MAE of 0.72%. These deviations agree with the overall errors shown in Fig. 4(b), where 30% and 100% ground-truth concentrations reported MAEs of 18.29% and 0.75%, respectively. Overall, the dual-wavelength atlas method achieved a greater accuracy when estimating concentration levels that primarily include MB.

Our initial dual-wavelength atlas method used LW-SLSC beamforming to extract meaningful photoacoustic data.^46^ The method herein uses M-Weighted SLSC beamforming, rather than LW-SLSC beamforming to reduce the extensive processing times required to generate coherence masks for a total of 2200 frames (i.e., 11 concentrations ×2 wavelengths ×10 frames ×5 trials ×2 datasets) of photoacoustic data. However, considering that only a single ultrasound image is needed to show the structural detail surrounding the concentration maps in Figs. 5 and 6, LW-SLSC was employed to beamform the background ultrasound image and improve the contrast, edges, and interpretation of the structural details surrounding the photoacoustic signals.

### 3.3 Effect of Mask Size on Concentration Estimation Performance

To illustrate the impact of mask size on estimation performance, coherence masks were generated from M-weighted SLSC images with coherence thresholds ranging from 0.3 to 0.9 in increments of 0.02. These masks were used to segment photoacoustic signals from the human blood dataset and generated concentration maps of different sizes. Then, for each coherence threshold, the MAE was calculated for the all 10 frames per 11 concentration levels.

**Fig. 7:**
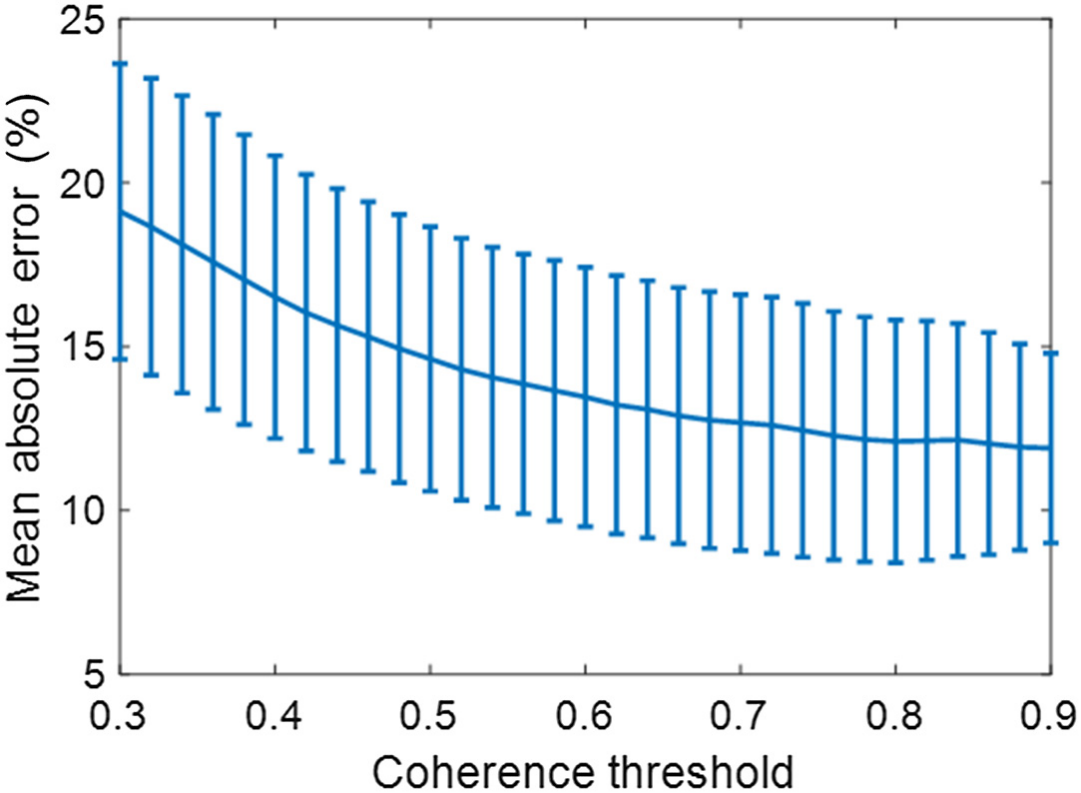
MAE of the estimated chromophore concentration obtained with the dual-wavelength atlas method as a function of M-weighted SLSC coherence thresholds.

Figure 7 shows the performance of the dual-wavelength atlas method when using segmented masks generated with varying coherence thresholds. Overall, the MAE decreases when using higher coherence thresholds to generate the segmentation masks. Therefore, we selected a coherence threshold of 0.7 to generate all coherence masks in the experiment results.

**Fig. 8:**
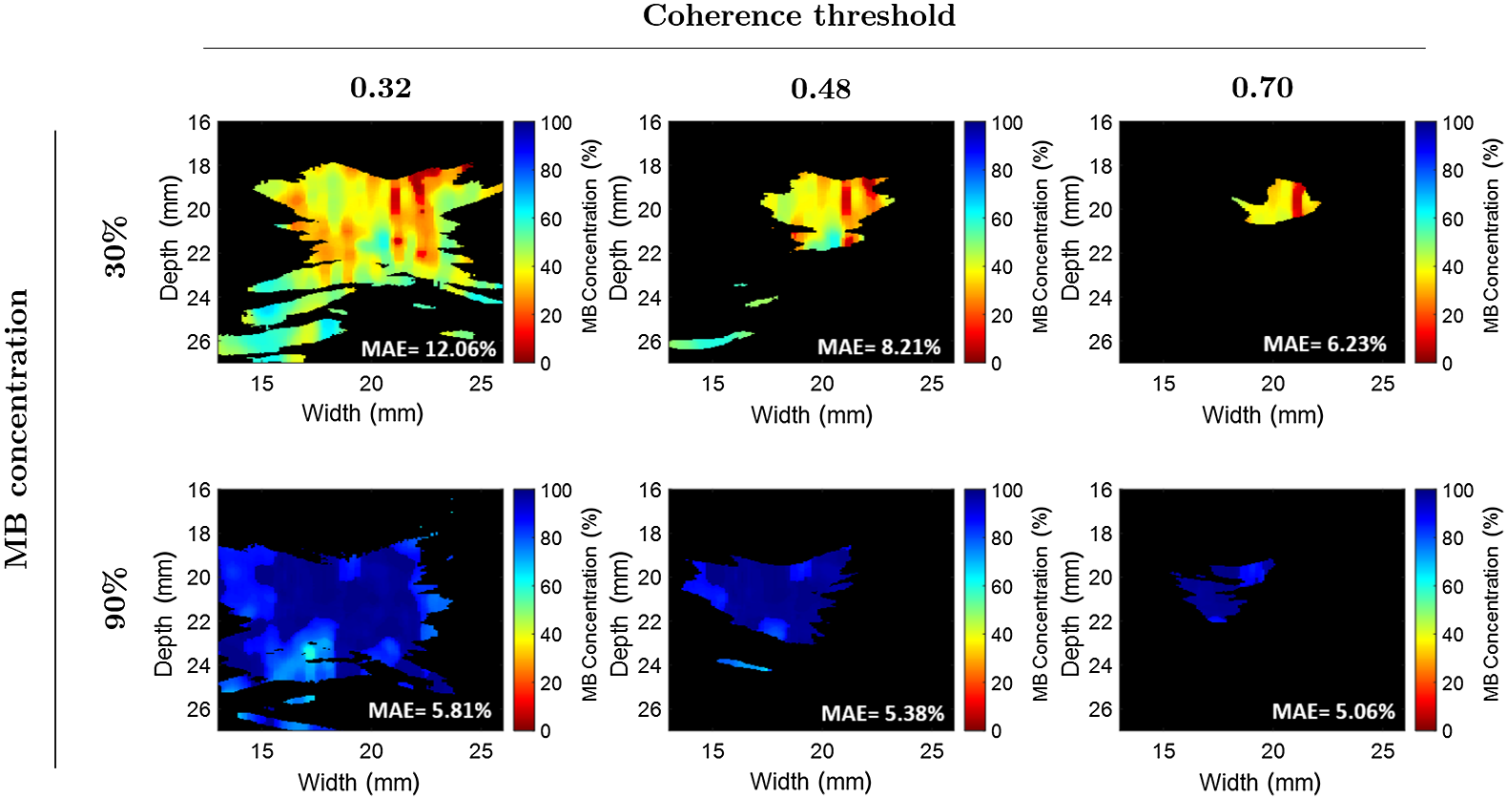
Example concentration maps generated with different coherence threshold values.

Figure 8 shows examples of concentration maps with a mixture of 30% MB (top) and 90% MB (bottom) when using coherence thresholds of 0.32, 0.48, and 0.70 (from left to right, respectively). The measured MAE are reported in the lower right corner of each image. For the 30% MB mixture, when increasing the coherence threshold from 0.32 to 0.70, a MAE decrease from 12.06% to 6.23% (i.e., 5.83% decrease) is observed. The reduced error is attributed to the absence of inaccurate estimates near the bottom of the 0.70 concentration map. In contrast, for the 90% MB mixture, when increasing the coherence threshold from 0.32 to 0.70, a MAE decrease of from 5.81% to 5.06% (i.e., 0.75% decrease) is observed, which can be attributed to a more uniform distribution of the mixture across the chamber. Overall, decreasing the mask size of the region of interest with more appropriate thresholding, increases the performance of the dual-wavelength atlas method, as observed in Figs. 7 and 8.

## 4 Discussion

We developed a novel photoacoustic-based, dual-wavelength atlas method that accurately estimates and generates concentration maps of a mixture of endogenous and exogenous chromophores (i.e., MB and Hb, respectively). The method builds on our previous dual-wavelength approach^46^ and measures the photoacoustic spectra obtained from two laser wavelength emissions as a linear combination of the chromophore spectra stored in an atlas. Linearity and monotonicity were confirmed with analyses of acoustic spectra and estimated concentrations as the ground-truth concentration increased, as shown in Figs. 3 and 4, respectively.

Method optimization [see Fig. 3(a)] resulted in requiring only the first principal component for feature reduction, which agrees with previous optimization results of the dual-wavelength atlas method.^46^^,^^66^ However, the number of principal components as well as the axial kernel size required for accurate estimation may increase with noise and diverse photoacoustic responses obtained from different factors, such as fiber tip geometries,^73^^–^^75^ vessel size,^76^^–^^78^ absorber size,^79^^–^^81^ *ex vivo*,^82^ and *in vivo*^83^^,^^84^ data. Future studies will investigate the effect of these factors in the optimization process of our method.

There are three main factors that contribute to the variability of the estimated concentration maps shown in Fig. 6. First, the energy fluctuations provided by the laser system translated into unequal fluence among frames (e.g., the mean ± one standard deviation of the laser energy at the fiber tip was 3.13±0.4  mJ for the human Hb experiment). Second, decreasing the coherence threshold chosen to segment meaningful photoacoustic signals when generating M-weighted SLSC masks decreased the estimation performance of the dual-wavelength atlas method, as shown in Figs. 7 and 8. Third, although precautions were taken to minimize differences in oxygen saturation among Hb samples, such variations were not negligible. Energy fluctuations among consecutive frames and differences in oxygen-saturation levels can be reduced when using fast-tuning lasers and fresh Hb from a single patient, respectively, in more realistic clinical scenarios.

When extending our approach to other ultrasound systems, implementation with a different transducer bandwidth would require an additional IQ calibration step to maximize chromophore concentration estimation performance. Typically, the IQ compression of radiofrequency signals is conducted using a modulation frequency equal to the central frequency of the ultrasound transducer. However, as observed in the stacked spectra shown in Fig. 2, most of the frequency content resides within 1 to 4 MHz, whereas the corresponding transducer center frequency is 5.5 MHz. Therefore, the modulation frequency and bandwidth parameter of the IQ modulation step should be adjusted^66^ to segment meaningful spectra from the total bandwidth of the transducer and thus enhance the estimation performance of the proposed algorithm.

*In vivo* deployment of the dual-wavelength atlas method for mixture estimation requires two considerations. First, given that constructing a photoacoustic atlas is relatively fast (i.e., <10  min), extracting presurgical Hb samples from a single patient would be beneficial for minimizing the estimation variability due to Hb samples from different patients at different draw times in the training set. Second, smaller vasculature are expected to yield different spectral responses because the frequency components are dependent on the volume of the chamber that is irradiated.^78^^,^^85^ For reference, the diameter of the PVCP chamber simulated the average size of the inferior vena cava (i.e., 0.46 to 2.26 cm in diameter^86^). Therefore, vessel-specific parameter optimization is a possible future direction that can be explored with various chamber diameters.

## 5 Conclusion

The work contained herein is the first to present an acoustic-based photoacoustic estimator that relies on training sets to estimate concentration levels from mixtures of photoacoustic-sensitive materials. The proposed method consisted of measuring the acoustic spectra obtained from two laser wavelength emissions as a linear combination of the chromophore spectra stored in an atlas. This linear combination assumption was confirmed with phantom experiments. In clinical practice, we envision dual excitation wavelengths illuminating the region of interest with a fast-tuning laser source, providing real-time labeling of photoacoustic-sensitive regions with a parallelized version of the algorithm. The results from the presented experiments are promising for real-time monitoring of the concentration of contrast agents in the operating room.

## References

[1] Xu H. et al., “Nanoscale optical probes for cellular imaging,” Chem. Soc. Rev. 43(8), 2650–2661 (2014). CSRVBR 0306-0012 10.1039/c3cs60309a 24394966

[2] Wu D. et al., “Contrast agents for photoacoustic and thermoacoustic imaging: a review,” Int. J. Mol. Sci. 15(12), 23616–23639 (2014).1422-0067 10.3390/ijms151223616 25530615 PMC4284784

[3] Moore C. Jokerst J. V., “Strategies for image-guided therapy, surgery, and drug delivery using photoacoustic imaging,” Theranostics 9(6), 1550 (2019). 10.7150/thno.32362 31037123 PMC6485201

[4] Xia J. Kim C. Lovell J. F, “Opportunities for photoacoustic-guided drug delivery,” Curr. Drug Targets 16(6), 571–581 (2015). 10.2174/1389450116666150707100328 26148989 PMC5435469

[5] Nie L. et al., “In vivo volumetric photoacoustic molecular angiography and therapeutic monitoring with targeted plasmonic nanostars,” Small 10(8), 1585–1593 (2014). SMALBC 1613-6810 10.1002/smll.201302924 24150920

[6] Mallidi S. Luke G. P. Emelianov S., “Photoacoustic imaging in cancer detection, diagnosis, and treatment guidance,” Trends Biotechnol. 29(5), 213–221 (2011). TRBIDM 0167-7799 10.1016/j.tibtech.2011.01.006 21324541 PMC3080445

[7] Wang X. et al., “Noninvasive photoacoustic angiography of animal brains in vivo with near-infrared light and an optical contrast agent,” Opt. Lett. 29(7), 730–732 (2004). OPLEDP 0146-9592 10.1364/OL.29.000730 15072373

[8] Kim C. et al., “Deeply penetrating *in vivo* photoacoustic imaging using a clinical ultrasound array system,” Biomed. Opt. Express 1(1), 278–284 (2010). BOEICL 2156-7085 10.1364/BOE.1.000278 21258465 PMC3005157

[9] Kim C. et al., “Performance benchmarks of an array-based hand-held photoacoustic probe adapted from a clinical ultrasound system for non-invasive sentinel lymph node imaging,” Philos. Trans. R. Soc. A: Math. Phys. Eng. Sci. 369(1955), 4644–4650 (2011). 10.1098/rsta.2010.0353 PMC3263783 22006911

[10] Ku G. Wang L. V., “Deeply penetrating photoacoustic tomography in biological tissues enhanced with an optical contrast agent,” Opt. Letters 30(5), 507–509 (2005). OPLEDP 0146-9592 10.1364/OL.30.000507 15789718

[11] Cardinell K. et al., “A novel photoacoustic-fluorescent contrast agent for quantitative imaging of lymphatic drainage,” Photoacoustics 21, 100239 (2021). 10.1016/j.pacs.2021.100239 33520651 PMC7820935

[12] Wang D. Wu Y. Xia J., “Review on photoacoustic imaging of the brain using nanoprobes,” Neurophotonics 3(1), 010901 (2016). 10.1117/1.NPh.3.1.010901 26740961 PMC4699324

[13] Pan D. et al., “Molecular photoacoustic imaging of angiogenesis with integrin-targeted gold nanobeacons,” FASEB J. 25(3), 875–882 (2011). FAJOEC 0892-6638 10.1096/fj.10-171728 21097518 PMC3042842

[14] Gu Y.-J. et al., “Nuclear penetration of surface functionalized gold nanoparticles,” Toxicol. Appl. Pharmacol. 237(2), 196–204 (2009). TXAPA9 0041-008X 10.1016/j.taap.2009.03.009 19328820

[15] Koo J. et al., “*In vivo* non-ionizing photoacoustic mapping of sentinel lymph nodes and bladders with ICG-enhanced carbon nanotubes,” Phys. Med. Biol. 57(23), 7853 (2012). PHMBA7 0031-9155 10.1088/0031-9155/57/23/7853 23151772

[16] Filonov G. S. et al., “Deep-tissue photoacoustic tomography of a genetically encoded near-infrared fluorescent probe,” Angew. Chem. 124(6), 1477–1480 (2012). ANCEAD 0044-8249 10.1002/ange.201107026 PMC3293502 22213541

[17] Razansky D. et al., “Multispectral opto-acoustic tomography of deep-seated fluorescent proteins *in vivo*,” Nat. Photonics 3(7), 412–417 (2009). NPAHBY 1749-4885 10.1038/nphoton.2009.98

[18] Jeon M. et al., “Methylene blue microbubbles as a model dual-modality contrast agent for ultrasound and activatable photoacoustic imaging,” J. Biomed. Opt. 19(1), 016005 (2014). JBOPFO 1083-3668 10.1117/1.JBO.19.1.016005 24390438

[19] Song K. H. et al., “Noninvasive photoacoustic identification of sentinel lymph nodes containing methylene blue *in vivo* in a rat model,” J. Biomed. Opt. 13(5), 054033 (2008). JBOPFO 1083-3668 10.1117/1.2976427 19021413 PMC2725003

[20] Rajian J. R. et al., “Drug delivery monitoring by photoacoustic tomography with an icg encapsulated double emulsion,” Opt. Express 19(15), 14335–14347 (2011). OPEXFF 1094-4087 10.1364/OE.19.014335 21934797 PMC3324934

[21] Thawani J. P. et al., “Photoacoustic-guided surgery with indocyanine green-coated superparamagnetic iron oxide nanoparticle clusters,” Small 13(37), 1701300 (2017). SMALBC 1613-6810 10.1002/smll.201701300 PMC5884067 28748623

[22] Cho W.-S. et al., “Acute toxicity and pharmacokinetics of 13 nm-sized PEG-coated gold nanoparticles,” Toxicol. Appl. Pharmacol. 236(1), 16–24 (2009). TXAPA9 0041-008X 10.1016/j.taap.2008.12.023 19162059

[23] Tsoli M. et al., “Cellular uptake and toxicity of Au_55_ clusters,” Small 1(8–9), 841–844 (2005). SMALBC 1613-6810 10.1002/smll.200500104 17193536

[24] Alkilany A. M. Murphy C. J., “Toxicity and cellular uptake of gold nanoparticles: what we have learned so far?” J. Nanopart. Res. 12(7), 2313–2333 (2010). JNARFA 1388-0764 10.1007/s11051-010-9911-8 21170131 PMC2988217

[25] Hope-Ross M. et al., “Adverse reactions due to indocyanine green,” Ophthalmology 101(3), 529–533 (1994). OPANEW 0743-751X 10.1016/S0161-6420(94)31303-0 8127574

[26] Salahuddin N. et al., “Antibacterial and cytotoxicity of methylene blue loaded-cellulose nanocarrier on breast cancer cell line,” Carbohydr. Polym. Technol. Appl. 2, 100138 (2021). 10.1016/j.carpta.2021.100138

[27] Au T. H. et al., “The prevention of contrast-induced nephropathy,” Ann. Pharmacother. 48(10), 1332–1342 (2014). 10.1177/1060028014541996 24994723

[28] Kim C. et al., “Sentinel lymph nodes and lymphatic vessels: noninvasive dual-modality in vivo mapping by using indocyanine green in rats—volumetric spectroscopic photoacoustic imaging and planar fluorescence imaging,” Radiology 255(2), 442–450 (2010). RADLAX 0033-8419 10.1148/radiol.10090281 20413757 PMC2858815

[29] Nogueira J. J. et al., “Impact of lipid environment on photodamage activation of methylene blue,” ChemPhotoChem 1(5), 178–182 (2017). 10.1002/cptc.201600062

[30] Zharov V. P. et al., “*In vivo* photoacoustic flow cytometry for monitoring of circulating single cancer cells and contrast agents,” Opt. Lett. 31(24), 3623–3625 (2006). OPLEDP 0146-9592 10.1364/OL.31.003623 17130924

[r31] Nie L. et al., “Palladium nanosheets as highly stable and effective contrast agents for in vivo photoacoustic molecular imaging,” Nanoscale 6(3), 1271–1276 (2014). NANOHL 2040-3364 10.1039/C3NR05468C 24317132

[32] Pan D. et al., “A brief account of nanoparticle contrast agents for photoacoustic imaging,” Wiley Interdiscip. Rev.: Nanomed. Nanobiotechnol. 5(6), 517–543 (2013). 10.1002/wnan.1231 23983210 PMC4067981

[33] Tzoumas S. Ntziachristos V., “Spectral unmixing techniques for optoacoustic imaging of tissue pathophysiology,” Philos. Trans. R. Soc. A: Math. Phys. Eng. Sci. 375(2107), 20170262 (2017). 10.1098/rsta.2017.0262 PMC5647272 29038385

[r34] Gröhl J. et al., “Estimation of blood oxygenation with learned spectral decoloring for quantitative photoacoustic imaging (LSD-qPAI),” arXiv:1902.05839 (2019).

[r35] Gröhl J. et al., “Learned spectral decoloring enables photoacoustic oximetry,” Sci. Rep. 11, 6565 (2021). SRCEC3 2045-2322 10.1038/s41598-021-83405-8 33753769 PMC7985523

[r36] Dahlstrand U. et al., “Photoacoustic imaging for three-dimensional visualization and delineation of basal cell carcinoma in patients,” Photoacoustics 18, 100187 (2020). 10.1016/j.pacs.2020.100187 32461885 PMC7243191

[r37] Arabul M. et al., “Unmixing multi-spectral photoacoustic sources in human carotid plaques using non-negative independent component analysis,” Photoacoustics 15, 100140 (2019). 10.1016/j.pacs.2019.100140 31417847 PMC6690666

[r38] Weber J. Beard P. C. Bohndiek S. E., “Contrast agents for molecular photoacoustic imaging,” Nat. Methods 13(8), 639–650 (2016).1548-7091 10.1038/nmeth.3929 27467727

[r39] Weissleder R. Ntziachristos V., “Shedding light onto live molecular targets,” Nat. Med. 9(1), 123–128 (2003).1078-8956 10.1038/nm0103-123 12514725

[40] Brunker J. et al., “Photoacoustic imaging using genetically encoded reporters: a review,” J. Biomed. Opt. 22(7), 070901 (2017). JBOPFO 1083-3668 10.1117/1.JBO.22.7.070901 28717818

[41] Lediju Bell M. A., “Photoacoustic imaging for surgical guidance: principles, applications, and outlook,” J. Appl. Phys. 128(6), 060904 (2020). JAPIAU 0021-8979 10.1063/5.0018190 32817994 PMC7428347

[r42] Wiacek A. et al., “Dual-wavelength photoacoustic imaging for guidance of hysterectomy procedures,” Proc. SPIE 11229, 112291D (2020). PSISDG 0277-786X 10.1117/12.2544906

[r43] Wiacek A. Bell M. A. L., “Photoacoustic-guided surgery from head to toe,” Biomed. Opt. Express 12(4), 2079–2117 (2021). BOEICL 2156-7085 10.1364/BOE.417984 33996218 PMC8086464

[44] Donnelly E. M. et al., “Photoacoustic image-guided delivery of plasmonic-nanoparticle-labeled mesenchymal stem cells to the spinal cord,” Nano Lett. 18(10), 6625–6632 (2018). NALEFD 1530-6984 10.1021/acs.nanolett.8b03305 30160124

[45] Glatz J. et al., “Blind source unmixing in multi-spectral optoacoustic tomography,” Opt. Express 19(4), 3175–3184 (2011). OPEXFF 1094-4087 10.1364/OE.19.003175 21369139

[46] Gonzalez E. A. Graham C. A. Lediju Bell M. A., “Acoustic frequency-based approach for identification of photoacoustic surgical biomarkers,” Front. Photonics 2, 716656 (2021). 10.3389/fphot.2021.716656

[47] Grasso V. Holthof J. Jose J., “An automatic unmixing approach to detect tissue chromophores from multispectral photoacoustic imaging,” Sensors 20(11), 3235 (2020). SNSRES 0746-9462 10.3390/s20113235 PMC7308815 32517204

[48] Xia W. et al., “Interventional multispectral photoacoustic imaging with a clinical linear array ultrasound probe for guiding nerve blocks,” Proc. SPIE 9708, 97080C (2016). PSISDG 0277-786X 10.1117/12.2209047

[49] Cao Y. et al., “Spectral analysis assisted photoacoustic imaging for lipid composition differentiation,” Photoacoustics 7, 12–19 (2017). 10.1016/j.pacs.2017.05.002 28649497 PMC5472148

[50] Moore M. J. et al., “Photoacoustic F-mode imaging for scale specific contrast in biological systems,” Commun. Phys. 2, 30 (2019). 10.1038/s42005-019-0131-y

[51] Jeevarathinam A. S. et al., “A cellulose-based photoacoustic sensor to measure heparin concentration and activity in human blood samples,” Biosens. Bioelectron. 126, 831–837 (2019). BBIOE4 0956-5663 10.1016/j.bios.2018.11.052 30602265 PMC6357780

[r52] Cash K. J. et al., “Optical drug monitoring: photoacoustic imaging of nanosensors to monitor therapeutic lithium *in vivo*,” ACS Nano 9(2), 1692–1698 (2015). ANCAC3 1936-0851 10.1021/nn5064858 25588028 PMC4364417

[53] Ho I.-T. et al., “Parts per billion detection of uranium with a porphyrinoid-containing nanoparticle and in vivo photoacoustic imaging,” Analyst 140(11), 3731–3737 (2015). ANLYAG 0365-4885 10.1039/C5AN00207A 25854506 PMC4437871

[54] Graham M. et al., “*In vivo* demonstration of photoacoustic image guidance and robotic visual servoing for cardiac catheter-based interventions,” IEEE Trans. Med. Imaging 39(4), 1015–1029 (2020). ITMID4 0278-0062 10.1109/TMI.2019.2939568 31502964

[55] Bui N. Q. et al., “Intravascular ultrasonic–photoacoustic (IVUP) endoscope with 2.2-mm diameter catheter for medical imaging,” Comput. Med. Imaging Graph. 45, 57–62 (2015). 10.1016/j.compmedimag.2015.07.008 26258625

[56] Raut S. Gaffney P., “Evaluation of the fibrin binding profile of two anti-fibrin monoclonal antibodies,” Thromb. Haemost. 76(1), 56–64 (1996). THHADQ 0340-6245 10.1055/s-0038-1650522 8819252

[57] De La Zerda A. et al., “Carbon nanotubes as photoacoustic molecular imaging agents in living mice,” Nat. Nanotechnol. 3(9), 557–562 (2008). NNAABX 1748-3387 10.1038/nnano.2008.231 18772918 PMC2562547

[58] Zerda A. D. L. et al., “Ultrahigh sensitivity carbon nanotube agents for photoacoustic molecular imaging in living mice,” Nano Lett. 10(6), 2168–2172 (2010). NALEFD 1530-6984 10.1021/nl100890d 20499887 PMC2893026

[59] Cheresh D. A., “Integrins in thrombosis, wound healing and cancer,” Biochem. Soc. Trans. 19(4), 835–838 (1991). BCSTB5 0300-5127 10.1042/bst0190835 1794568

[60] Sowers T. Emelianov S., “Exogenous imaging contrast and therapeutic agents for intravascular photoacoustic imaging and image-guided therapy,” Phys. Med. Biol. 63(22), 22TR01 (2018). PHMBA7 0031-9155 10.1088/1361-6560/aae62b 30403195

[61] Wagner S. J. et al., “Factors affecting virus photoinactivation by a series of phenothiazine dyes,” Photochem. Photobiol. 67(3), 343–349 (1998). PHCBAP 0031-8655 10.1111/j.1751-1097.1998.tb05208.x 9523534

[r62] Jack Clifton I. Leikin J. B., “Methylene blue,” Am. J. Ther. 10(4), 289–291 (2003). 10.1097/00045391-200307000-00009 12845393

[r63] Ginimuge P. R. Jyothi S., “Methylene blue: revisited,” J. Anaesthesiol. Clin. Pharmacol. 26(4), 517 (2010). 10.4103/0970-9185.74599 21547182 PMC3087269

[64] Buchholz K. et al., “Interactions of methylene blue with human disulfide reductases and their orthologues from plasmodium falciparum,” Antimicrob. Agents Chemother. 52(1), 183–191 (2008). 10.1128/AAC.00773-07 17967916 PMC2223905

[65] Gonzalez E. A. Bell M. A. L., “Acoustic frequency-based differentiation of photoacoustic signals from surgical biomarkers,” in IEEE International Ultrasonics Symposium (IUS), IEEE, pp. 1–4 (2020). 10.1109/IUS46767.2020.9251551

[66] Gonzalez E. A. Graham C. A. Bell M. A. L., “Optimization of a dual wavelength atlas technique to differentiate methylene blue from hemoglobin in photoacoustic signals,” Proc. SPIE 11960, 119600A (2022). 10.1117/12.2607825

[67] Nair A. A. Tran T. D. Bell M. A. L., “Robust short-lag spatial coherence imaging,” IEEE Trans. Ultrason. Ferroelectr. Freq. Control 65(3), 366–377 (2018). ITUCER 0885-3010 10.1109/TUFFC.2017.2780084 29505405 PMC5870140

[68] Gonzalez E. A. Bell M. A. L., “GPU implementation of photoacoustic short-lag spatial coherence imaging for improved image-guided interventions,” J. Biomed. Opt. 25(7), 077002 (2020). JBOPFO 1083-3668 10.1117/1.JBO.25.7.077002 PMC7381831 32713168

[69] Guindon B. Zhang Y., “Application of the dice coefficient to accuracy assessment of object-based image classification,” Can. J. Remote Sens. 43(1), 48–61 (2017). CJRSDP 0703-8992 10.1080/07038992.2017.1259557

[70] Gonzalez E. A. Jain A. Bell M. A. L., “Combined ultrasound and photoacoustic image guidance of spinal pedicle cannulation demonstrated with intact *ex vivo* specimens,” IEEE Trans. Biomed. Eng. 68(8), 2479–2489 (2021). IEBEAX 0018-9294 10.1109/TBME.2020.3046370 33347403 PMC8345233

[71] Sprent P., Applied Nonparametric Statistical Methods, Springer Science & Business Media (2012).

[72] Schiks I. et al., “Performance evaluation of arterial femoral sheath removal by registered nurses after pci,” Eur. J. Cardiovasc. Nurs. 6(3), 172–177 (2007). 10.1016/J.EJCNURSE.2006.08.001 16997633

[73] Gonzalez E. Wiacek A. Bell M. A. L., “Visualization of custom drill bit tips in a human vertebra for photoacoustic-guided spinal fusion surgeries,” Proc. SPIE 10878, 108785M (2019). PSISDG 0277-786X 10.1117/12.2510688

[r74] Eddins B. Bell M. A. L., “Design of a multifiber light delivery system for photoacoustic-guided surgery,” J. Biomed. Opt. 22(4), 041011 (2017). JBOPFO 1083-3668 10.1117/1.JBO.22.4.041011 PMC5995140 28114443

[75] Shubert J. Bell M. A. L., “A novel drill design for photoacoustic guided surgeries,” Proc. SPIE 10494, 104940J (2018). PSISDG 0277-786X 10.1117/12.2291247

[76] Zhang M. et al., “Photoacoustic power azimuth spectrum for microvascular evaluation,” Photoacoustics 22, 100260 (2021). 10.1016/j.pacs.2021.100260 33777693 PMC7985563

[r77] Jiang Y. et al., “Photoacoustic and high-frequency power Doppler ultrasound biomicroscopy: a comparative study,” J. Biomed. Opt. 15(5), 056008 (2010). JBOPFO 1083-3668 10.1117/1.3491126 21054102

[78] Kolkman R. G. et al., “Real-time *in vivo* photoacoustic and ultrasound imaging,” J. Biomed. Opt. 13(5), 050510 (2008). JBOPFO 1083-3668 10.1117/1.3005421 19021380

[79] Hysi E. et al., “Insights into photoacoustic speckle and applications in tumor characterization,” Photoacoustics 14, 37–48 (2019). 10.1016/j.pacs.2019.02.002 31080733 PMC6505056

[r80] Gamelin J. K. et al., “Curved array photoacoustic tomographic system for small animal imaging,” J. Biomed. Opt. 13(2), 024007 (2008). JBOPFO 1083-3668 10.1117/1.2907157 18465970 PMC2507725

[81] Strohm E. M. Kolios M. C., “Classification of blood cells and tumor cells using label-free ultrasound and photoacoustics,” Cytometry Part A 87(8), 741–749 (2015). 10.1002/cyto.a.22698 26079610

[82] Daeichin V. et al., “Frequency analysis of the photoacoustic signal generated by coronary atherosclerotic plaque,” Ultrasound Med. Biol. 42(8), 2017–2025 (2016). USMBA3 0301-5629 10.1016/j.ultrasmedbio.2016.03.015 27181689

[83] Kumon R. E. Deng C. X. Wang X., “Frequency-domain analysis of photoacoustic imaging data from prostate adenocarcinoma tumors in a murine model,” Ultrasound Med. Biol. 37(5), 834–839 (2011). USMBA3 0301-5629 10.1016/j.ultrasmedbio.2011.01.012 21376447 PMC3060609

[84] Feng T. et al., “Ultrasound-guided detection and segmentation of photoacoustic signals from bone tissue in vivo,” Appl. Sci. 11(1), 19 (2021). 10.3390/app11010019

[85] Diebold G. Sun T. Khan M., “Photoacoustic monopole radiation in one, two, and three dimensions,” Phys. Rev. Lett. 67(24), 3384 (1991). PRLTAO 0031-9007 10.1103/PhysRevLett.67.3384 10044720

[86] Patil S. et al., “Assessment of inferior vena cava diameter by echocardiography in normal Indian population: a prospective observational study,” Indian Heart J. 68, S26–S30 (2016). 10.1016/j.ihj.2016.06.009 28038721 PMC5198879

